# Cost-effectiveness of ramucirumab plus paclitaxel as maintenance therapy for patients with advanced HER2-negative gastric or gastroesophageal junction cancer

**DOI:** 10.3389/fonc.2026.1713870

**Published:** 2026-02-09

**Authors:** Dong Li, Xue Zhao, Huixian Jia, Rong Cui, Afang Cao, Liman Huo

**Affiliations:** 1 Department of Pharmacy, The Fourth Hospital of Hebei Medical University, Shijiazhuang, China; 2 Department of Endocrinology, The Second Hospital of Hebei Medical University, Shijiazhuang, China

**Keywords:** cost-effectiveness analysis, gastric cancer, Markov model, pharmacoeconomics, ramucirumab

## Abstract

**Objective:**

This study a to evaluate the cost-effectiveness of ramucirumab plus paclitaxel as a switch maintenance therapy for patients with advanced HER2-negative gastric or gastroesophageal junction cancer, from the perspective of the Chinese healthcare system, to guide clinical decision-making and reimbursement policies.

**Methods:**

A three-state Markov model (progression-free, progressed disease, and death) was developed based on data from the ARMANI phase III randomized controlled trial. Two treatment strategies were compared: ramucirumab plus paclitaxel versus continuation of oxaliplatin-based chemotherapy (FOLFOX/CAPOX). Cost data were obtained from national procurement platforms and published literature; utility values were derived from EQ-5D instruments. A willingness-to-pay (WTP) was set at three times China’s 2024 per capita GDP, equivalent to USD 40, 457.32 per QALY.Base-case analysis, one-way and probabilistic sensitivity analyses, threshold price analysis, and a patient assistance program (PAP) scenario were conducted.

**Results:**

The base-case analysis yielded an incremental cost-effectiveness ratio (ICER) of USD 138, 320.14/QALY, which exceeded the WTP threshold. Under the PAP scenario (i.e., “buy two, get one free”), the ICER was reduced to USD 95, 304.89/QALY, but remained above the threshold. Sensitivity analyses showed the model was most sensitive to utility values, discount rates, and drug price. Threshold analysis suggested that the price of ramucirumab would need to be reduced to approximately 24% of its current level (~USD 149/100mg) to achieve cost-effectiveness; even under the PAP scenario, a further price cut to 36% (~USD 223/100 mg) would still be required.

**Conclusion:**

At current pricing and under the existing PAP framework, ramucirumab plus paclitaxel is not a cost-effective switch maintenance strategy in advanced HER2-negative gastric or gastroesophageal junction cancer in China. Significant price reductions through national negotiations may enable this regimen to become economically viable within the Chinese healthcare system.

## 1 Introduction

Gastric cancer remains one of the leading causes of cancer-related mortality worldwide, with over one million new cases diagnosed annually (1). For patients with advanced HER2-negative gastric or gastroesophageal junction cancer, current clinical guidelines recommend fluoropyrimidine-based oxaliplatin chemotherapy as first-line treatment (2). However, the median progression-free survival (PFS) associated with this regimen is only approximately 6 months, and overall survival (OS) rarely exceeds 12 months (3). Furthermore, due to rapid disease progression, limits access to later lines of anticancer therapy to only 40–50% of patients (4), consequently highlighting the urgent need to optimize initial treatment strategies to prolong disease control and improve outcomes.

Although recent studies have explored triplet chemotherapy or immunotherapy-based regimens in selected subgroups—such as patients with high PD-L1 expression or mismatch repair deficiency (5)—most HER2-negative patients with low PD-L1 expression still lack effective first-line or maintenance treatment options. The ARMANI trial introduced the concept of “switch maintenance” therapy, proposing the use of ramucirumab plus paclitaxel following induction chemotherapy to consolidate response and delay disease progression (6).

Despite demonstrating clinical survival benefits in trial settings, the high cost of ramucirumab may hinder its widespread adoption in the Chinese healthcare system. As drug reimbursement policies and pricing negotiations continue to evolve, there is a pressing need for localized pharmacoeconomic evaluations to assess both clinical value and cost-effectiveness.

Therefore, based on data from the ARMANI phase III trial, this study constructed a three-state Markov model to evaluate the long-term cost-effectiveness of ramucirumab plus paclitaxel as switch maintenance therapy in advanced HER2-negative gastric or gastroesophageal junction cancer, from the perspective of the Chinese healthcare system. The goal is to provide quantitative evidence to support both clinical pathway optimization and national reimbursement decision-making.

## 2 Materials and methods

### 2.1 Target population

The target population in this study was defined based on the inclusion and exclusion criteria of the phase III multicenter randomized controlled ARMANI trial (6).

Inclusion criteria were as follows:(1) age ≥18 years;(2) Eastern Cooperative Oncology Group (ECOG) performance status of 0–1;(3) an expected survival of at least 12 weeks; (4) histologically confirmed gastric or gastroesophageal junction adenocarcinoma at an unresectable locally advanced or metastatic stage; (5) human epidermal growth factor receptor 2 (HER2) negative status; (6) presence of measurable or evaluable lesions as defined by RECIST version 1.1; (7) completion of 3 months of induction chemotherapy with oxaliplatin plus fluoropyrimidine (six cycles of FOLFOX or four cycles of CAPOX); patients with measurable lesions were required to have achieved complete response (CR), partial response (PR), or stable disease (SD), while those without measurable lesions should show no disease progression; (8) adequate bone marrow, hepatic, and renal function.

Exclusion criteria included:(1) relapse within 12 months after prior adjuvant chemotherapy containing platinum and fluoropyrimidine; (2) history of arterial thromboembolic events within the past 6 months or venous thromboembolism within 3 months; (3) ongoing treatment with full-dose anticoagulants (e.g., warfarin, low molecular weight heparin, or Xa inhibitors; aspirin ≤325 mg/day was allowed); (4) history of bleeding disorders, vasculitis, gastrointestinal perforation or fistula, or clinically significant gastrointestinal bleeding within the past 3 months; (5) uncontrolled hypertension; (6) unresolved adverse events from prior chemotherapy, especially grade ≥2 oxaliplatin-related peripheral neuropathy.

### 2.2 Treatment strategies

A total of 280 patients were enrolled in this study and randomly assigned in a 1:1 ratio to either the intervention group (ramucirumab plus paclitaxel as switch maintenance therapy, n = 144) or the control group (continuation of platinum-based doublet chemotherapy, n = 136). All patients had completed first-line induction chemotherapy, which consisted of six cycles of the FOLFOX regimen (oxaliplatin 85 mg/m², leucovorin 400 mg/m², fluorouracil 400 mg/m² as intravenous bolus, followed by a 46-hour continuous infusion of fluorouracil 2400 mg/m², every 2 weeks) or four cycles of the CAPOX regimen (oxaliplatin 130 mg/m² plus oral capecitabine 1000 mg/m² twice daily on days 1–14, every 3 weeks).

In the intervention group (switch maintenance arm), each treatment cycle lasted 28 days. Paclitaxel 80 mg/m² was administered intravenously on days 1, 8, and 15 of each cycle, and ramucirumab 8 mg/kg was given intravenously on days 1 and 15. Treatment continued until one of the following occurred: disease progression, intolerable toxicity, withdrawal of informed consent, investigator decision to discontinue treatment, or death.

In the control group, patients continued the same oxaliplatin-based induction regimen—either FOLFOX (administered every 2 weeks) or CAPOX (administered every 3 weeks)—for up to an additional 12 weeks, resulting in a maximum cumulative duration of 24 weeks of oxaliplatin-containing therapy. Oxaliplatin was mandatorily discontinued at week 24. Patients without disease progression at that time proceeded to a fluoropyrimidine monotherapy maintenance phase, consisting of either: (i) intravenous 5-fluorouracil (400 mg/m² bolus + 2400 mg/m² continuous infusion over 46 hours) with leucovorin (400 mg/m²), administered every 2 weeks; or (ii) oral capecitabine (1000 mg/m² twice daily on days 1–14 of each 21-day cycle).

All patients were followed up every 12 weeks until death or withdrawal from the study.

### 2.3 Model structure

A Markov model with three mutually exclusive health states—progression-free survival (PFS), progressive disease (PD), and death—was developed to evaluate the long-term cost-effectiveness of the treatment strategies. The primary clinical inputs for the model were derived from the ARMANI Phase III randomized controlled trial (6). All patients were assumed to enter the model in the PFS state and could transition between health states in each cycle based on predefined transition probabilities.

**Figure 1:**
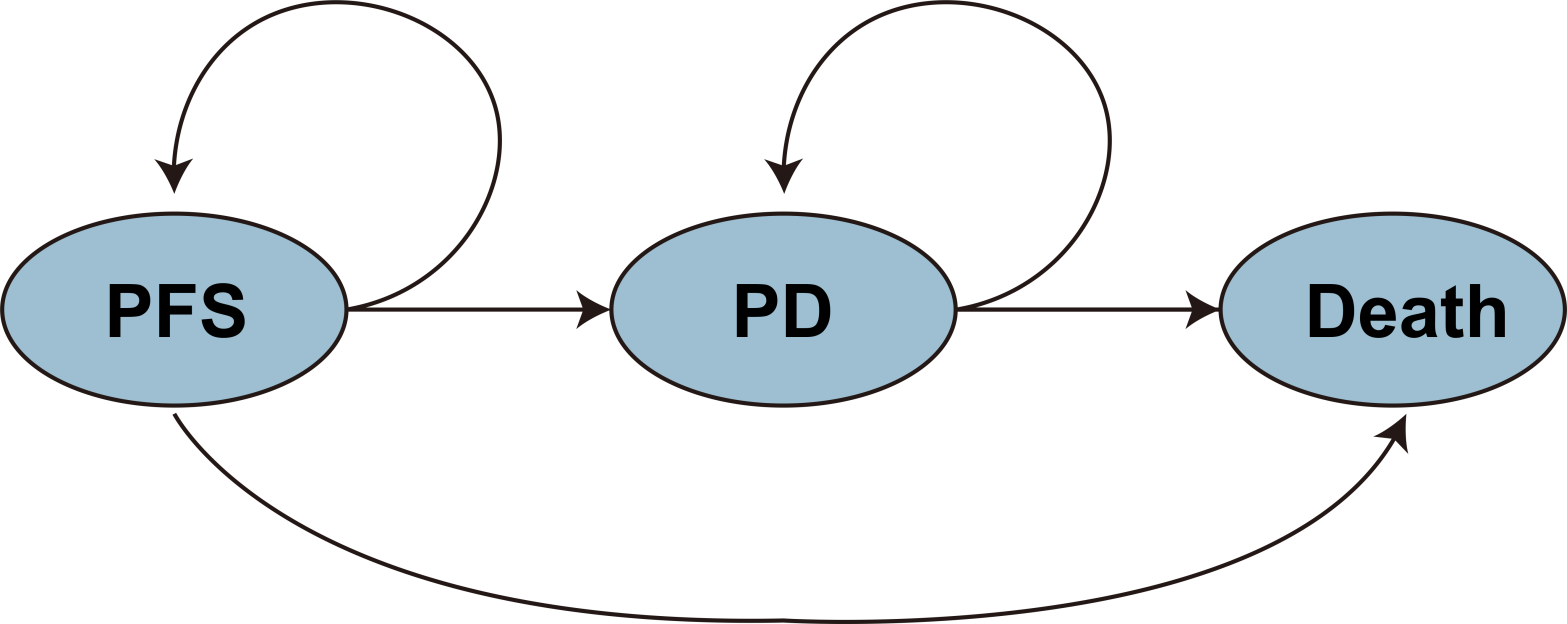
Structure of the three-state Markov model.

The economic model was implemented in R (version 4.4.2; R Core Team, 2024), and its structure is shown in **Figure 1**. A 10-year time horizon was adopted, with a cycle length of 4 weeks (28 days), resulting in a total of 130 cycles. The model outputs included total cost, quality-adjusted life years (QALYs), and the incremental cost-effectiveness ratio (ICER).

In the base-case analysis, the willingness-to-pay (WTP) threshold was set at three times China’s gross domestic product (GDP) per capita in 2024, equivalent to 40, 457.32 USD/QALY (7). Following the recommendations of the Chinese Guidelines for Pharmacoeconomic Evaluations (2020 edition), both costs and utilities werediscounted at an annual rate of 5% (8).

### 2.4 Survival modeling and estimation

In this study, survival data for the intention-to-treat population were extracted from the ARMANI Phase III trial using the WebPlotDigitizer software. Progression-free survival (PFS) and overall survival (OS) curves were digitized, and individual patient-level data (IPD) were reconstructed using the algorithm proposed by Guyot et al. (27). Modeling and extrapolation were performed in R version 4.4.0.

To identify the best-fitting survival curves, 15 commonly used parametric and flexible models were fitted to the reconstructed data. These included traditional parametric distributions (Exponential, Weibull, Gamma, Log-normal, Gompertz, Log-logistic, Generalized Gamma), flexible models (Fractional Polynomial [FP1, FP2], Restricted Cubic Splines [RCS], and Royston-Parmar models with hazard, odds, or normal scales), mixture-cure models (MCM), and generalized additive models (GAM).

Model fit was assessed using the Akaike Information Criterion (AIC) and Bayesian Information Criterion (BIC), complemented by visual inspection to determine the most appropriate extrapolation method (9). Survival functions were derived from the optimal models, which were then used to estimate transition probabilities between PFS to PD and PD to death across Markov cycles (10).

**Figure 2:**
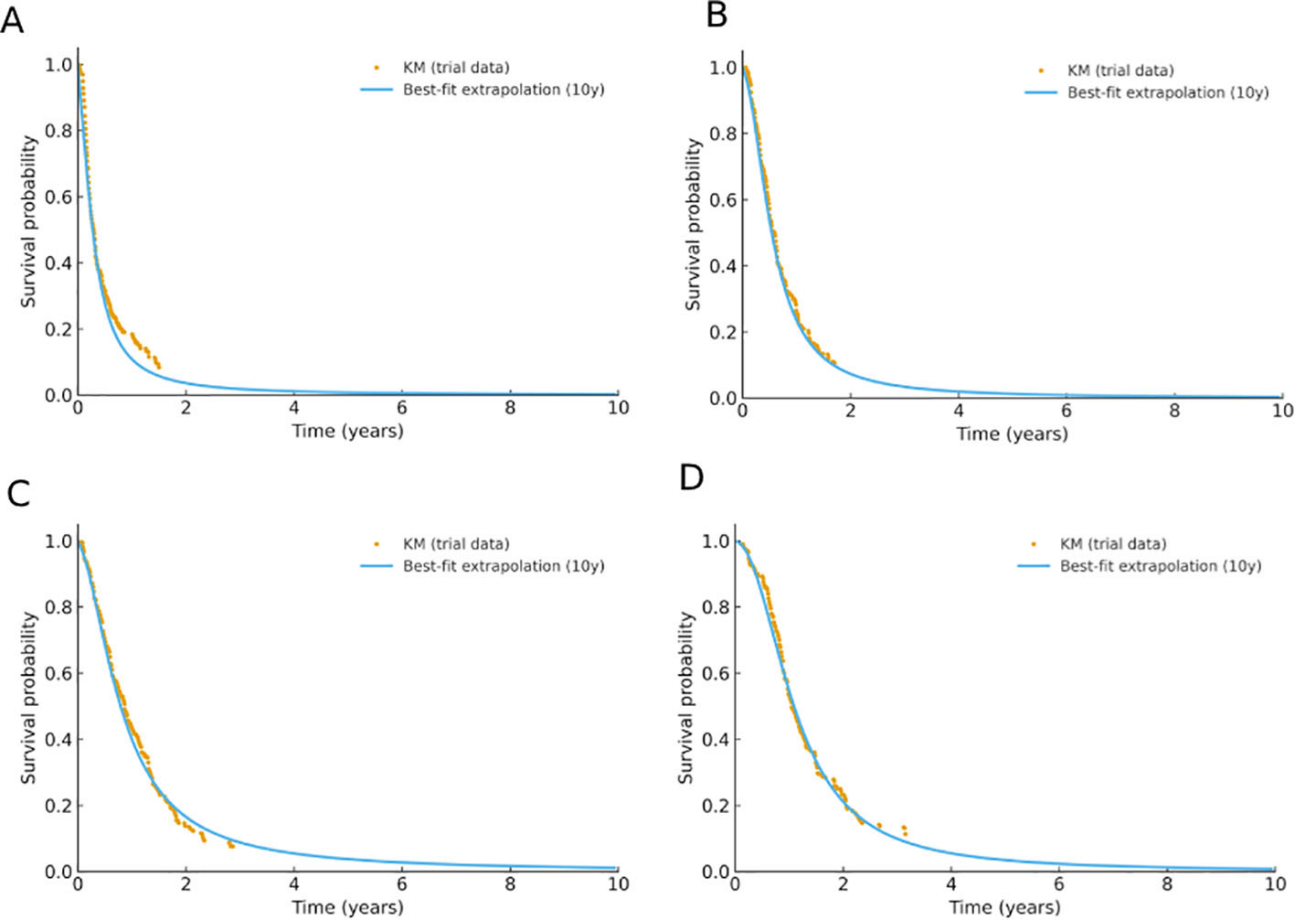
Fitting and Extrapolation Results of PFS Curves **(A, B)** and OS Curves **(C, D)**. In **(A)** and **(B)** PFS_CG and PFS_TG represent the progression-free survival (PFS) fitted curves for the control group (CG) and treatment group (TG), respectively. The mix-cure distribution model was applied to the control group, while the RP-normal model was used for the treatment group. In **(C)** and **(D)** OS_CG and OS_TG represent the overall survival (OS) fitted curves for the two groups, with the FP1 distribution model applied to the control group and the FP2 model to the treatment group. Dashed lines indicate the survival curves fitted based on actual observed data, while solid lines represent the extrapolated survival curves derived from the distribution models. TG Paclitaxel-Ramucirumab Treatment Group; CG Control Group (receiving FOLFOX or CAPOX therapy).

Among all candidate models, those with the minimum AIC and BIC values were considered to have the best fit. The optimal survival models for each treatment arm were as follows: in the control group, OS and PFS were modeled using FP1 and a mixture-cure model, respectively; in the intervention group, OS was fitted with an FP2 model, and PFS with a Royston-Parmar (normal scale) model. Detailed parameter estimates are presented in **Table 1**, and the fitted survival curves are illustrated in **Figure 2**.

**Table 1:** Goodness-of-fit test results for survival distribution models.

Model	OS curve				PFS curve			
	Paclitaxel-ramucirumab group		Maintenance treatment group		Paclitaxel-ramucirumab group		Maintenance treatment group	
	**AIC**	**BIC**	**AIC**	**BIC**	**AIC**	**BIC**	**AIC**	**BIC**
Exponential	169.34	172.31	131.58	134.55	113.53	116.49	106.10	109.07
Weibull	149.48	155.42	125.66	131.60	101.44	107.38	108.35	114.29
Gamma	142.66	148.60	123.70	129.64	96.86	102.80	107.16	113.10
Log-normal	135.47	141.41	122.92	128.86	89.83	95.77	84.99	90.93
Gompertz	165.60	171.54	131.02	136.96	111.28	117.22	105.65	111.59
Log-logistic	133.02	138.96	123.22	129.16	90.97	96.91	88.67	94.61
Generalized Gamma	137.60	146.51	123.48	132.38	92.13	101.04	77.73	86.64
FP1	139.41	145.35	**120.42**	**126.36**	90.63	96.57	93.89	99.83
FP2	**129.62**	**138.53**	121.93	130.84	90.55	99.46	74.23	83.14
RCS	132.93	144.81	127.48	136.39	96.73	108.60	76.92	91.77
RP-hazard	133.72	145.60	123.91	132.82	92.03	100.94	78.79	87.70
RP-odds	133.30	145.18	123.22	129.16	90.97	96.91	78.44	87.35
RP-normal	133.46	145.34	123.52	132.43	**89.83**	**95.77**	82.67	97.52
GAM	133.39	146.03	127.29	135.65	96.58	108.01	76.83	91.40
MCM	285.84	294.75	245.93	254.84	164.47	173.38	**47.23**	**62.08**

AIC and BIC values for the best-fitting model are highlighted in bold red.

### 2.5 Cost and utility data

From the perspective of the Chinese healthcare system, this study included only direct medical costs related to the treatment strategies. These costs comprised drug acquisition, laboratory and imaging examinations, best supportive care, and the management of grade ≥3 adverse events (AEs) with an incidence of ≥2%. Cost data were primarily obtained from the Hebei Provincial Drug Procurement Platform and published literature sources (11–20).

The incidence of adverse events was based on data from the ARMANI phase III clinical trial, including neutropenia, anemia, peripheral neuropathy, and venous thromboembolism (6). To better reflect the anthropometric profile of Chinese patients with advanced gastric or gastroesophageal junction (GEJ) cancer, we estimated a reference body weight and height using nationally representative, sex-disaggregated data from the Report on the Nutrition and Chronic Disease Status of Chinese Residents (2020) (21). This estimate was adjusted for age-related height loss in older adults (22) and weighted according to the gender distribution in the ARMANI trial (64% male, 36% female) (6), yielding a reference weight of 66.9 kg and height of 163.5 cm. Using the Mosteller formula, this corresponds to a baseline body surface area (BSA) of 1.74 m².

Given the high prevalence of cancer cachexia in patients with advanced gastric or gastroesophageal junction (GEJ) adenocarcinoma—reported in 70% to 85% of cases—and consistent with international consensus criteria defining cachexia as non-volitional weight loss of ≥5% over six months, we did not assume that patients maintained their pre-illness reference body weight. Instead, the base-case analysis assumed a 10% weight loss (i.e., 90% of reference weight), corresponding to a body surface area (BSA) of 1.65 m², which reflects moderate cachexia commonly observed in patients eligible for second-line or maintenance therapy and is supported by clinical studies in similar populations (23–26). To account for greater severity of wasting, a worst-case scenario assuming 15% weight loss (BSA = 1.61 m²) was evaluated in sensitivity analyses. All drug doses and associated costs were calculated using these patient-specific BSA estimates rather than general-population averages, thereby mitigating potential overestimation of treatment costs due to unadjusted anthropometric assumptions.

For health utility values, the utility scores for progression-free survival (PFS) and progressive disease (PD) health states were assumed to be 0.68 and 0.42, respectively, derived from published studies (17). In the ARMANI trial, health-related quality of life was measured using the EQ-5D-3L instrument and converted using the UK value set. Detailed cost and utility parameters for each health state are presented in **Table 2**.

**Table 2:** Markov model parameters and distributions.

Parameter	Mean	Lower bound	Upper bound	Distribution	Source
FOLFOX cost per 28-day cycle	86.40	64.80	108.00	Gamma	Local data
Paclitaxel + Ramucirumab cost per 28-day cycle	6011.10	4508.33	7513.88	Gamma	Local data
Post-progression (treatment arm) FOLFIRI cost per 28-day cycle	149.83	112.37	187.29	Gamma	Local data
5fu cost per 28-day cycle	8.80	6.60	11.00	Gamma	Local data
Post-progression (treatment arm) Trifluridine–Tipiracil cost per 28-day cycle	552.68	414.51	690.85	Gamma	Local data
Post-progression (treatment arm) Best supportive care cost per 28-day cycle	211.27	158.45	264.08	Gamma	Local data
Laboratory tests cost	44.7	33.52	55.87	Gamma	Reference 11
Imaging cost	95.45	71.59	119.31	Gamma	Reference 11
Neutropenia management cost	321.67	241.25	402.08	Gamma	Reference 12
Anemia management cost	472.73	354.55	590.91	Gamma	Reference 13
Peripheral neuropathy management cost	3000	2250	3750	Gamma	Reference 14
Venous thromboembolism (VTE) management cost	9247	6935.25	11558.75	Gamma	Reference 15
End-of-life care cost	1419.15	1064.37	1773.94	Gamma	Reference 16
Neutropenia risk (intervention arm)	0.26	0.2	0.32	Beta	Reference 6
Neutropenia risk (control arm)	0.09	0.068	0.11	Beta	Reference 6
Anemia risk (intervention arm)	0.02	0.015	0.025	Beta	Reference 6
Anemia risk (control arm)	0.03	0.02	0.04	Beta	Reference 6
Peripheral neuropathy risk (intervention arm)	0.06	0.045	0.075	Beta	Reference 6
Peripheral neuropathy risk (control arm)	0.07	0.0525	0.0875	Beta	Reference 6
VTE risk (intervention arm)	0.03	0.0225	0.0375	Beta	Reference 6
VTE risk (control arm)	0	0	0	Beta	Reference 6
Utility: progression-free state (PFS)	0.68	0.51	0.85	Beta	Reference 17
Utility: post-progression state (PD)	0.42	0.315	0.525	Beta	Reference 17
Utility decrement: neutropenia	0.09	0.0675	0.1125	Beta	Reference 18
Utility decrement: anemia	0.125	0.09375	0.15625	Beta	Reference 18
Utility decrement: peripheral neuropathy	0.03	0.02	0.04	Beta	Reference 19
Utility decrement: VTE	0.08	0.06	0.09	Beta	Reference 20
Discount rate	0.05	0	0.08	Beta	Reference 8
Post-progression (treatment arm) regimen mix: FOLFIRI proportion	0.36	0.27	0.45	Beta	Reference 6
Post-progression (treatment arm) regimen mix: Trifluridine–Tipiracil proportion	0.17	0.1275	0.2125	Beta	Reference 6
Post-progression (treatment arm) regimen mix: Best supportive care proportion	0.29	0.2175	0.3625	Beta	Reference 6
Post-progression (treatment arm) regimen mix: FOLFOX proportion	0.18	0.135	0.225	Beta	Reference 6
Post-progression (control arm) regimen mix: Paclitaxel + Ramucirumab proportion	0.258	0.1935	0.3225	Beta	Reference 6
Post-progression (control arm) regimen mix: FOLFIRI proportion	0.112	0.084	0.14	Beta	Reference 6
Post-progression (control arm) regimen mix: Best supportive care proportion	0.63	0.4725	0.7875	Beta	Reference 6

### 2.6 Sensitivity analysis

To assess the robustness of the model results, one-way sensitivity analysis (OWSA) and probabilistic sensitivity analysis (PSA) were conducted in accordance with established pharmacoeconomic guidelines.

In the OWSA, key cost parameters (including drug acquisition, diagnostic, and follow-up costs) were varied independently within ±25% of their base-case values, a range commonly used to reflect plausible uncertainty in cost inputs when detailed variance data are unavailable. The impact on the incremental cost-effectiveness ratio (ICER) was summarized using a tornado diagram.

Utility values were varied based on reported uncertainty; published confidence intervals or standard errors were used when available, and otherwise a ±25% variation was applied, with all utility values constrained between 0 and 1. Transition probabilities were derived from parametric survival models, and uncertainty was incorporated by varying model parameters within their 95% confidence intervals rather than applying arbitrary percentage changes.

For the PSA, probabilistic sensitivity analysis was performed using Monte Carlo simulation with 1, 000 iterations. Cost parameters were assigned Gamma distributions, while utility values and transition probabilities were assigned Beta distributions. The results were presented using cost-effectiveness scatter plots and cost-effectiveness acceptability curves (CEACs), illustrating the probability of cost-effectiveness across a range of willingness-to-pay thresholds.

### 2.7 Scenario analysis

Given the high cost of ramucirumab (USD 616.05 per 100 mg vial), which is currently not included in the National Reimbursement Drug List (NRDL) or centralized procurement catalog in China, patients face a substantial out-of-pocket burden. To improve drug accessibility, the China Primary Health Care Foundation has launched a Patient Assistance Program (PAP).

The PAP adopts a “buy two, get one free” scheme, in which patients who complete two treatment cycles at their own expense can apply—pending program approval—for one additional cycle of free medication. This assistance can be repeatedly applied for until disease progression or treatment discontinuation. In this scenario analysis, it was assumed that all patients met the eligibility criteria for PAP, and the impact of this program on the cost-effectiveness of ramucirumab plus paclitaxel was evaluated accordingly.

In light of the high prevalence of cancer cachexia in patients with advanced gastric or gastroesophageal junction (GEJ) adenocarcinoma, we conducted a supplementary scenario analysis assuming a 15% reduction from pre-illness reference body weight, corresponding to a body surface area (BSA) of 1.61 m². This worst-case anthropometric assumption represents a clinically plausible upper bound of disease-related wasting and was employed to evaluate the robustness of cost estimates under more severe malnutrition. All drug doses and associated costs were recalculated using this reduced BSA to assess its potential impact on the incremental cost-effectiveness ratio (ICER).

## 3 Base-case analysis results

The economic evaluation indicated that the ramucirumab plus paclitaxel strategy yielded an additional 0.39 quality-adjusted life years (QALYs) compared to maintenance chemotherapy, at an incremental cost of USD 53, 342.45. The resulting incremental cost-effectiveness ratio (ICER) was USD 138, 320.14 per QALY (**Table 3**).

**Table 3:** Base-case analysis results.

Group	Total cost (USD)	Effect (QALYs)	Incremental cost (USD)	Incremental effect (QALYs)	ICER (USD/QALY)
Treatment Group	58, 186.72	1.14	53, 342.45	0.39	138, 320.14
Control Group	4, 844.27	0.75	–	–	–

Treatment group included patients who received paclitaxel plus ramucirumab, Control group included patients who continued with maintenance chemotherapy.

This study adopted three times the projected gross domestic product (GDP) per capita in China for 2024 (USD 13, 485.77 × 3 = USD 40, 457.32 per QALY) as the willingness-to-pay (WTP) threshold. Since the ICER exceeded the WTP threshold, the ramucirumab plus paclitaxel regimen was not considered cost-effective under current economic conditions in China.

### 3.1 One-way sensitivity analysis

**Figure 3:**
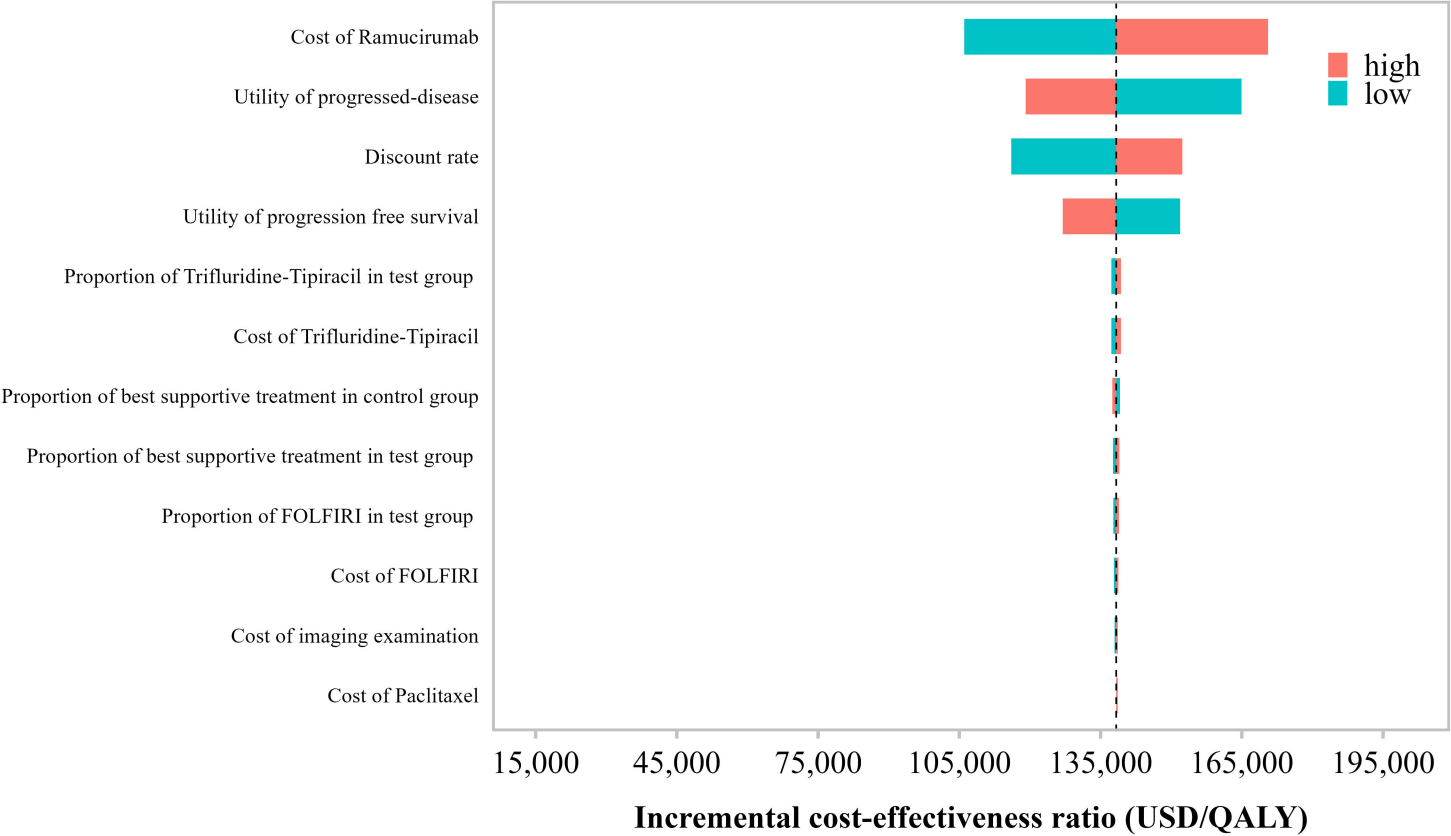
Tornado diagram of the one-way sensitivity analysis in the base-case scenario.

**Figure 3** presents the results of the one-way sensitivity analysis. The parameters with the greatest impact on the incremental cost-effectiveness ratio (ICER) were, in descending order: the cost of ramucirumab, utility value during the progressive disease (PD) state, discount rate, utility value during the progression-free survival (PFS) state, and the proportion of patients receiving paclitaxel plus ramucirumab in the control group. Other parameters showed relatively minor influences on the ICER.

Across the entire range of variation for each parameter, the ICER consistently remained above the predefined willingness-to-pay (WTP) threshold of USD 40, 457.32 per QALY, suggesting that the paclitaxel plus ramucirumab strategy was not cost-effective under any single-parameter variation.

### 3.2 Probabilistic sensitivity analysis results

**Figure 4:**
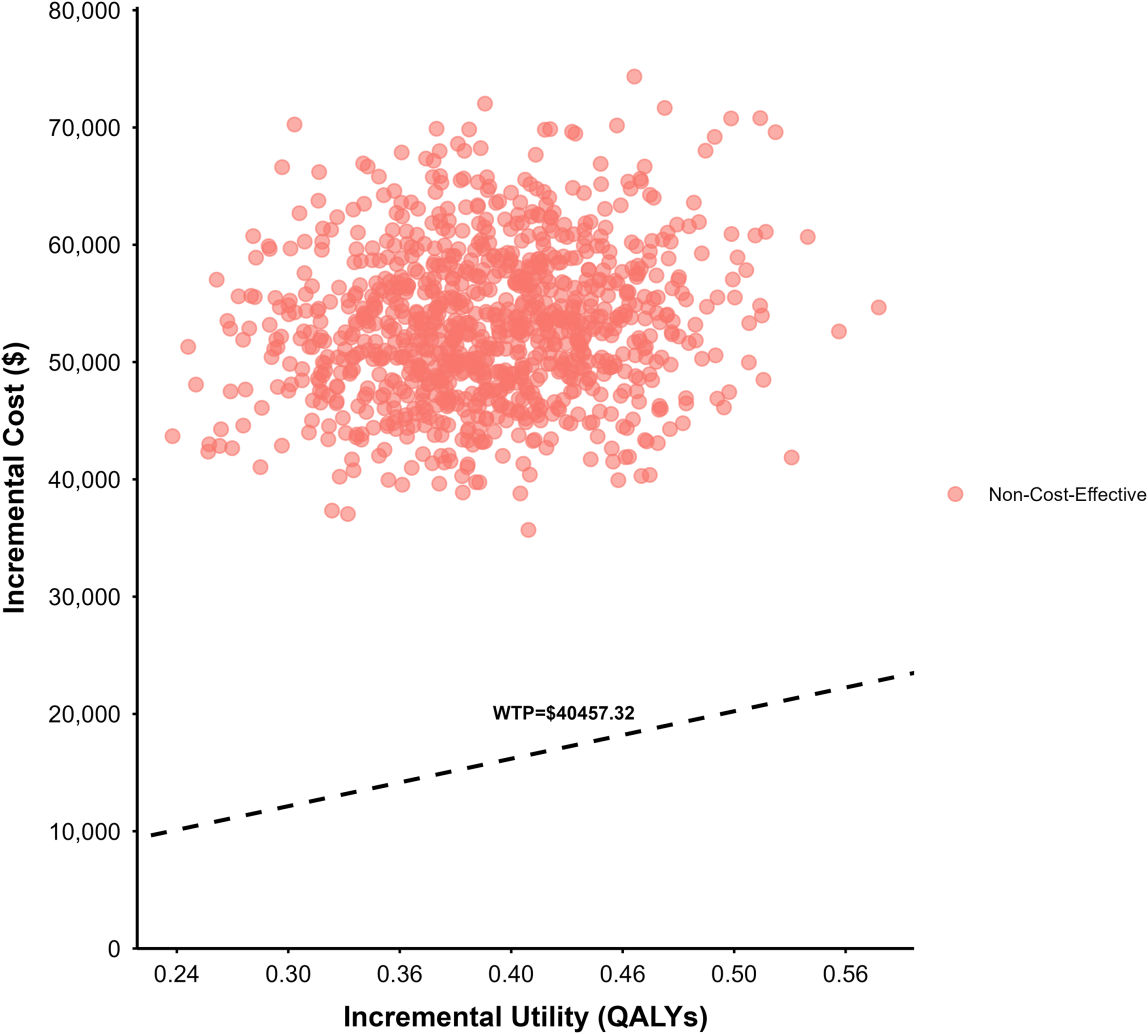
Cost-effectiveness scatter plot of the base-case analysis.

**Figure 5:**
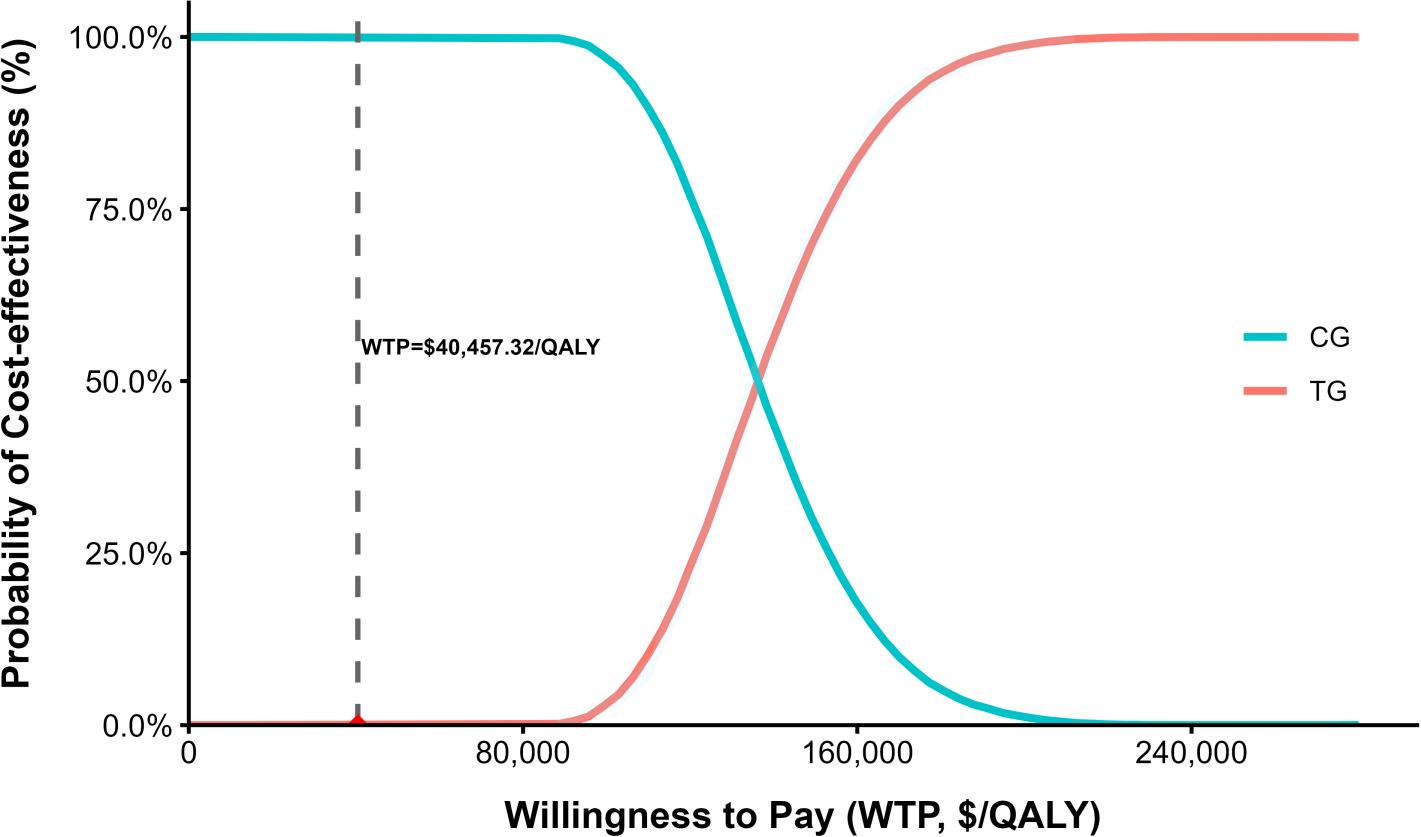
Cost-effectiveness acceptability curve (CEAC) of the base-case analysis.

The results of the probabilistic sensitivity analysis are presented in **Figure 4** (cost-effectiveness scatter plot) and **Figure 5** (cost-effectiveness acceptability curve).

As shown in **Figure 4**, all 1, 000 Monte Carlo simulations yielded incremental cost-effectiveness ratios (ICERs) above the willingness-to-pay (WTP) threshold of 40, 457.32 USD/QALY, indicating that the paclitaxel plus ramucirumab strategy is not cost-effective under most uncertainty scenarios. **Figure 5** further demonstrates that at a WTP threshold of 40, 457.32 USD/QALY, the probability of cost-effectiveness for the paclitaxel plus ramucirumab strategy is 0, reinforcing the conclusion that this treatment is not economically favorable under current cost and effectiveness assumptions.

### 3.3 Scenario analysis

Under the Patient Assistance Program (PAP), the total treatment cost of the paclitaxel plus ramucirumab regimen was substantially reduced, resulting in a decreased incremental cost-effectiveness ratio (ICER) of 95, 304.89 USD/QALY (**Table 4**). Although this value is lower than the base-case ICER of 138, 320.14 USD/QALY, it still exceeds the predefined willingness-to-pay (WTP) threshold of 40, 457.32 USD/QALY (three times China’s per capita GDP in 2024). Therefore, even with PAP support, the paclitaxel plus ramucirumab strategy does not demonstrate cost-effectiveness under current economic assumptions.

**Table 4:** Scenario analysis results.

Group	Total cost (USD)	Effect (QALYs)	Incremental cost (USD)	Incremental effect (QALYs)	ICER (USD/QALY)
Treatment Group	41, 598.11	1.14	36, 753.84	0.39	95, 304.89
Control Group	4, 844.27	0.75			

**Figure 6:**
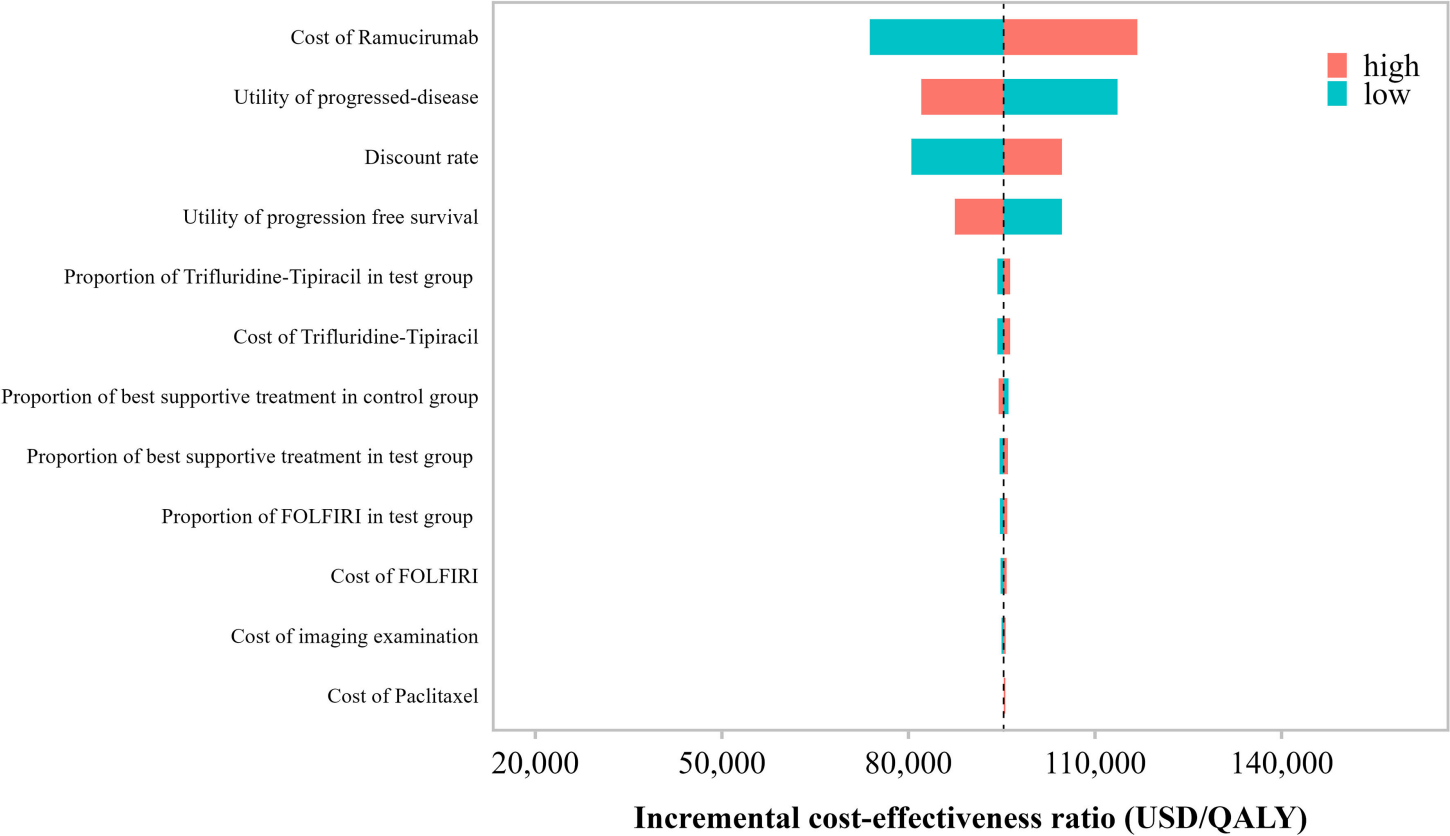
Tornado diagram of the one-way sensitivity analysis under the scenario analysis.

As shown in **Figure 6**, the one-way sensitivity analysis under the Patient Assistance Program (PAP) scenario identified the utility value in the progressive disease (PD) state, discount rate, utility value in the progression-free survival (PFS) state, and the proportion of patients in the control group receiving paclitaxel plus ramucirumab as the key drivers influencing the incremental cost-effectiveness ratio (ICER). Other parameters had relatively minor impacts.

**Figure 7:**
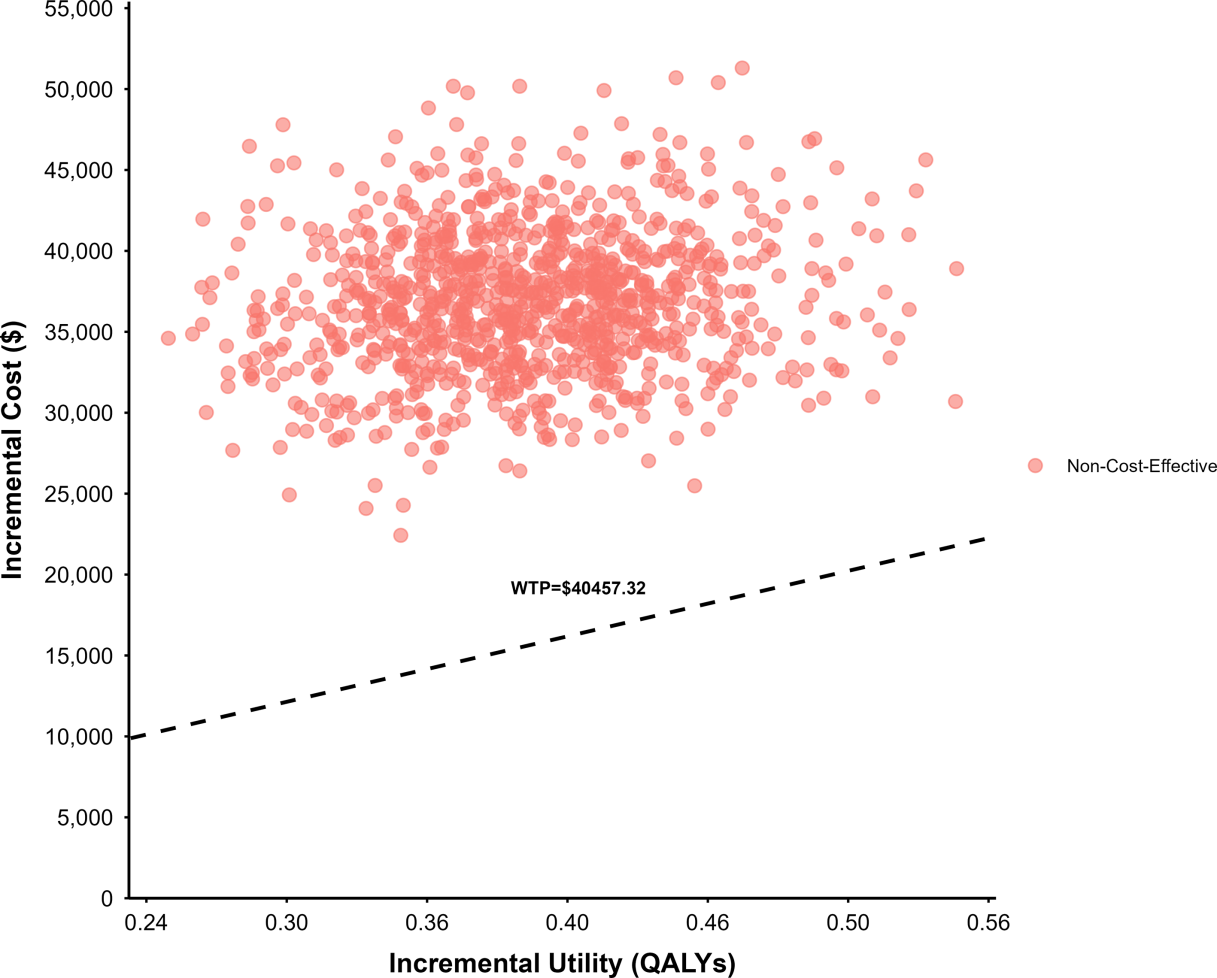
Cost-effectiveness scatter plot under the scenario analysis.

**Figure 8:**
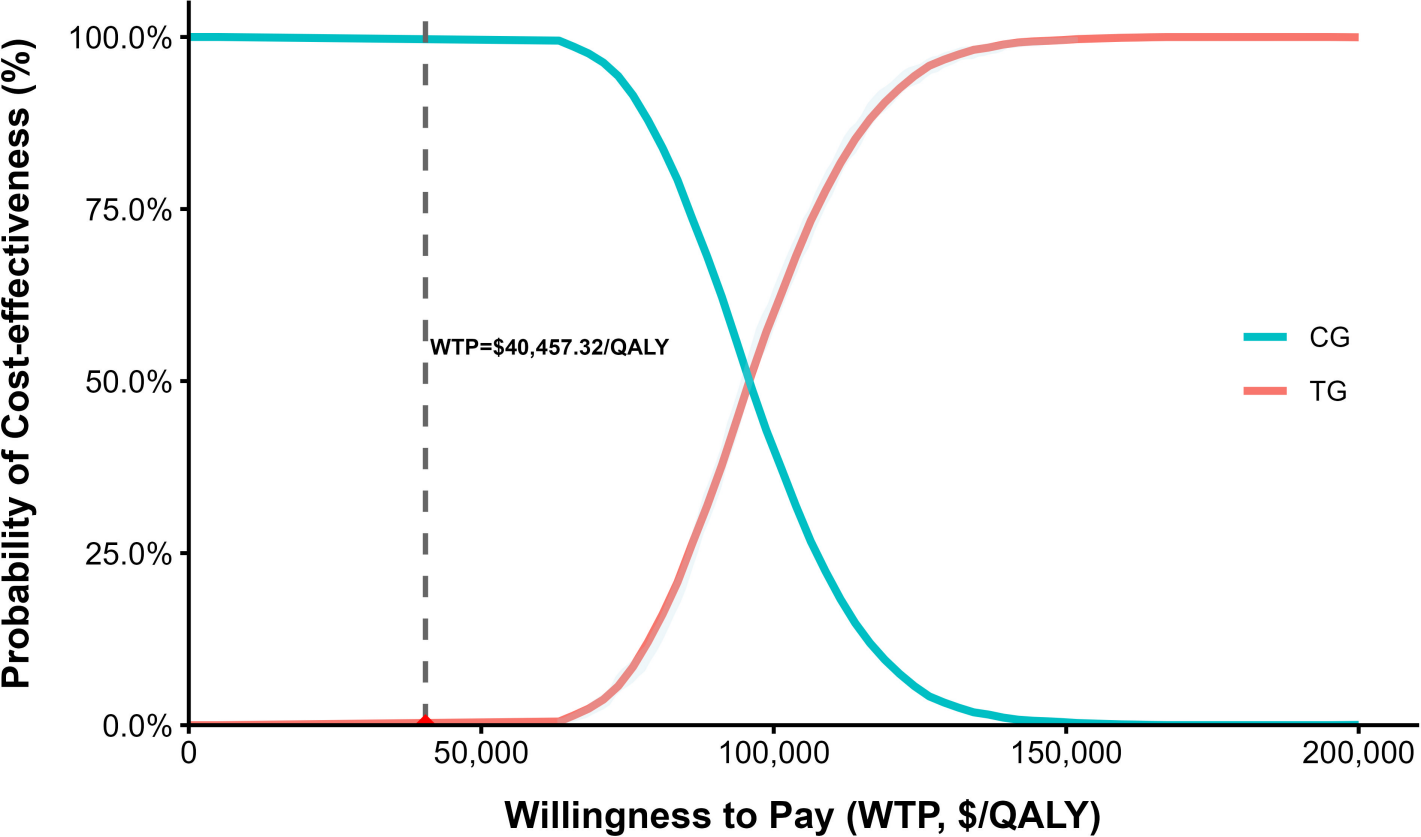
Cost-effectiveness acceptability curve under the scenario analysis.

**Figures 7** and **8** present the cost-effectiveness scatter plot and cost-effectiveness acceptability curve (CEAC), respectively. The results from 1, 000 Monte Carlo simulations showed that all ICER estimates fell above the willingness-to-pay (WTP) threshold of 40, 457.32 USD/QALY, indicating that the combination strategy of paclitaxel and ramucirumab was not cost-effective under most probabilistic scenarios. Furthermore, the CEAC revealed a 0% probability of cost-effectiveness at the current WTP threshold, reinforcing the conclusion that this treatment strategy lacks economic viability under the current assumptions.

In the scenario assuming 15% weight loss (BSA = 1.61 m²), the incremental cost-effectiveness ratio (ICER) for paclitaxel plus ramucirumab versus continued oxaliplatin-based chemotherapy was USD 131, 062.90 per QALY gained(**Table 5**), which substantially exceeds the predefined willingness-to-pay (WTP) threshold of USD 40, 457.32 per QALY. Thus, under this severe cachexia assumption, the switch maintenance strategy is not considered cost-effective.

**Table 5:** Scenario analysis results assuming a 15% weight reduction.

Group	Total cost (USD)	Effect (QALYs)	Incremental cost (USD)	Incremental effect (QALYs)	ICER (USD/QALY)
Treatment Group	55, 369.47	1.14	50, 543.73	0.39	131, 062.90
Control Group	4, 825.74	0.75			

### 3.4 Threshold analysis

The threshold analysis suggested that, using three times China’s per capita gross domestic product (GDP) as the willingness-to-pay (WTP) threshold, the maximum acceptable incremental cost would be USD 40, 457.32.

**Figure 9:**
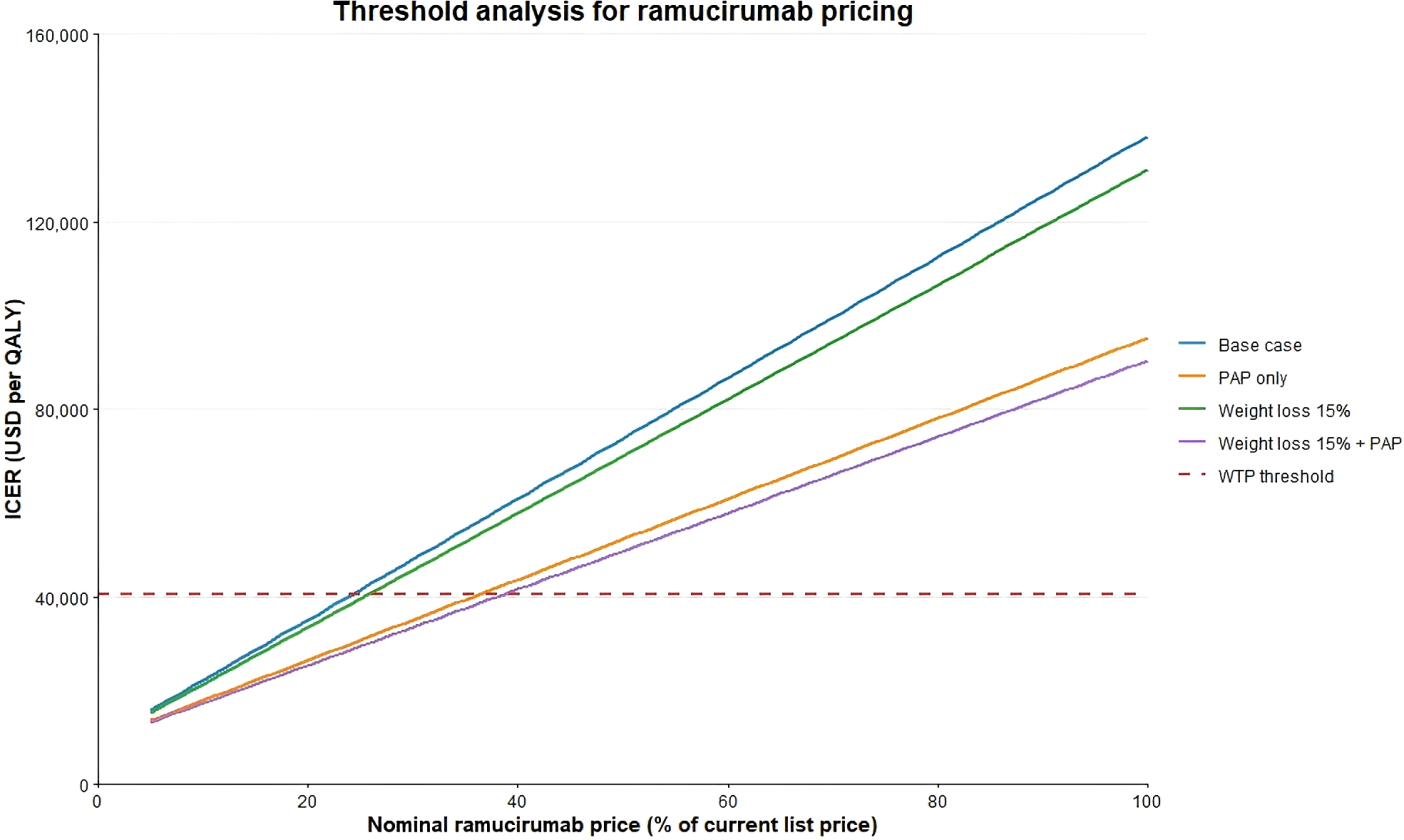
Threshold Analysis of Ramucirumab Price. The x-axis represents the proportion of ramucirumab price relative to the current nominal price, while the y-axis shows the incremental cost-effectiveness ratio (ICER, USD/QALY). The dashed line indicates the willingness-to-pay (WTP) threshold (40, 457.32 USD/QALY).

However, the observed incremental cost in this study was approximately 53, 342.45 USD. To make the treatment strategy cost-effective under this threshold, the unit price of ramucirumab would need to be reduced to about 24% of its current nominal price (approximately 149 USD/100 mg). Even under the “buy-two-get-one-free” Patient Assistance Program (PAP) scenario, the nominal drug price would still need to be further reduced to around 36% (approximately 223 USD/100 mg) for the ICER to fall below the WTP threshold (**Figure 9**).

## 4 Discussion

Although ramucirumab plus paclitaxel as a conversion maintenance strategy demonstrated clinically meaningful benefits in prolonging PFS in patients with advanced HER2-negative gastric or gastroesophageal junction cancer, our economic evaluation indicates that these clinical gains do not translate into cost-effectiveness under the current Chinese healthcare setting. This divergence between clinical efficacy and economic value highlights an important challenge in applying conversion maintenance strategies in resource-constrained health systems (6–8).

The lack of cost-effectiveness observed in our analysis is primarily driven by the intrinsic economic characteristics of the conversion maintenance strategy. Unlike finite-duration induction chemotherapy, ramucirumab-based maintenance therapy entails prolonged and continuous administration of a high-cost monoclonal antibody. Although this strategy extends disease control, it substantially increases cumulative drug expenditure over time, while the incremental QALY gain remains relatively modest. Consequently, the additional PFS benefit is insufficient to offset the long-term cost burden, resulting in an unfavorable ICER (6–8, 13).

Previous economic evaluations of ramucirumab-based regimens have largely focused on the second-line setting, particularly in the context of the RAINBOW trial. In contrast, the present study extends the evaluation to a conversion maintenance strategy initiated after first-line induction therapy. This distinction is economically relevant, as maintenance strategies inherently prolong treatment exposure, amplifying the impact of drug price on overall cost-effectiveness. Therefore, cost-effectiveness findings from second-line, time-limited treatment settings may not be directly applicable to conversion maintenance approaches (6, 13).

Although the PAP partially mitigates drug costs through policies such as “buy two, get one free, “ our results suggest that such schemes are insufficient to achieve cost-effectiveness for long-term maintenance therapy. Threshold analysis indicates that substantial price reductions would be required for ramucirumab to meet commonly accepted WTP thresholds in China. These findings underscore that assistance programs alone may have limited impact for high-cost biologics when treatment duration is prolonged, highlighting the need for structural price negotiations or value-based pricing (7, 8).

Several limitations should be acknowledged. First, clinical efficacy inputs were derived from a global randomized controlled trial rather than real-world data from Chinese populations, which may limit generalizability. Second, indirect costs were not incorporated, potentially underestimating the overall economic burden. Third, only grade ≥3 adverse events with an incidence ≥2% were included, which may overlook some low-frequency but clinically relevant events. Finally, treatment adherence in real-world practice may be lower than that observed in clinical trials, which could further influence cost-effectiveness outcomes (6, 11, 12).

Despite these limitations, extensive sensitivity, scenario, and threshold analyses consistently demonstrated the robustness of our findings. Collectively, this study provides quantitative evidence to support decision-making regarding pricing, reimbursement, and clinical implementation of conversion maintenance strategies in advanced gastric cancerand gastroesophageal junction cancer (6–8).

## 5 Conclusion

In conclusion, ramucirumab plus paclitaxel as conversion maintenance therapy improves clinical outcomes in advanced HER2-negative gastric or gastroesophageal junction cancer but is not cost-effective under current pricing and assistance schemes in China. Substantial drug price reductions or refined patient selection would be required for this strategy to meet accepted cost-effectiveness thresholds. These findings provide important evidence to inform future price negotiations and reimbursement decisions.

## Data Availability

The original contributions presented in the study are included in the article/supplementary material. Further inquiries can be directed to the corresponding author.
